# A Unique Case of Pili Multigemini Complicated by Folliculitis: Unveiling the Intricacies of a Rare Hair-Follicle Disorder

**DOI:** 10.7759/cureus.42280

**Published:** 2023-07-21

**Authors:** Sahil Navlani, Akshata Mestha

**Affiliations:** 1 General Practice, Dubai Academic Health Corporation, Dubai, ARE

**Keywords:** hair shaft dysplasia, hair disorders, folliculitis, dermoscopy, pili multigemini

## Abstract

Pili multigemini is defined as the presence of two or more hair shafts in one hair follicle. It has mostly been reported to occur in the beard of men; however, it has been reported to occur uncommonly at other locations of the body as well. We present a rare case of a patient who presented with folliculitis on the abdomen which was incidentally detected as pili multigemini.

## Introduction

Pili multigemini is a rare developmental disorder characterized by the presence of multiple hair shafts that arise from a single pilosebaceous canal and emerge from a single ostium. The hair shafts have a common outer root sheath, however, each shaft has an independent matrix, papilla, and inner root sheath [1]. It occurs more frequently in males, especially those with thick hair. They may present with recurrent inflammatory lesions which can have residual atrophic or hypertrophic scars. The most common location of occurrence is the beard along the jawlines, but it has been found in all regions of the body [2]. The prevalence rate has not been established and it has only been incidentally detected in 2% of patients who were symptomatic for other dermatological diseases [3]. They are usually benign and asymptomatic that do not require removal other than for cosmetic reasons [4].

## Case presentation

**Figure 1:**
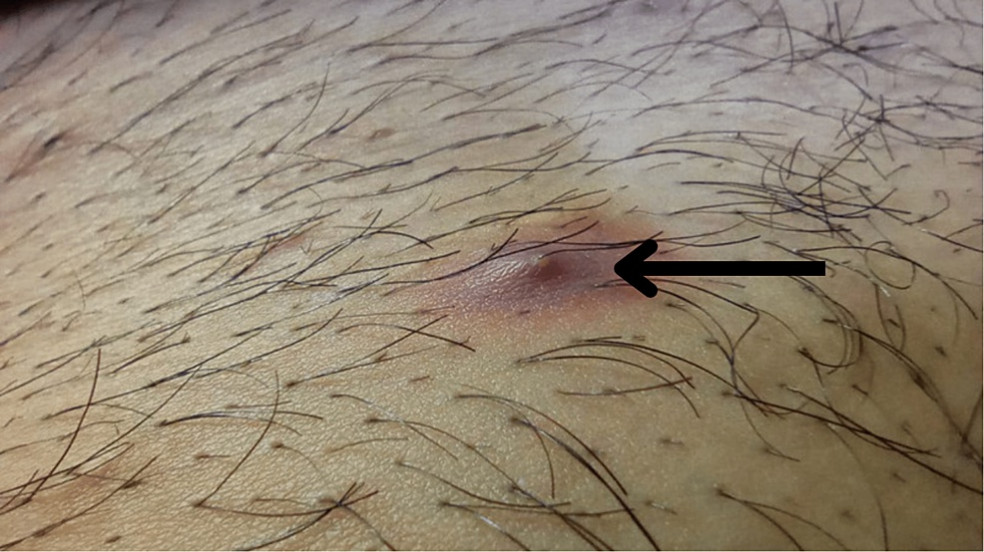
Folliculitis with pili multigemini

**Figure 2:**
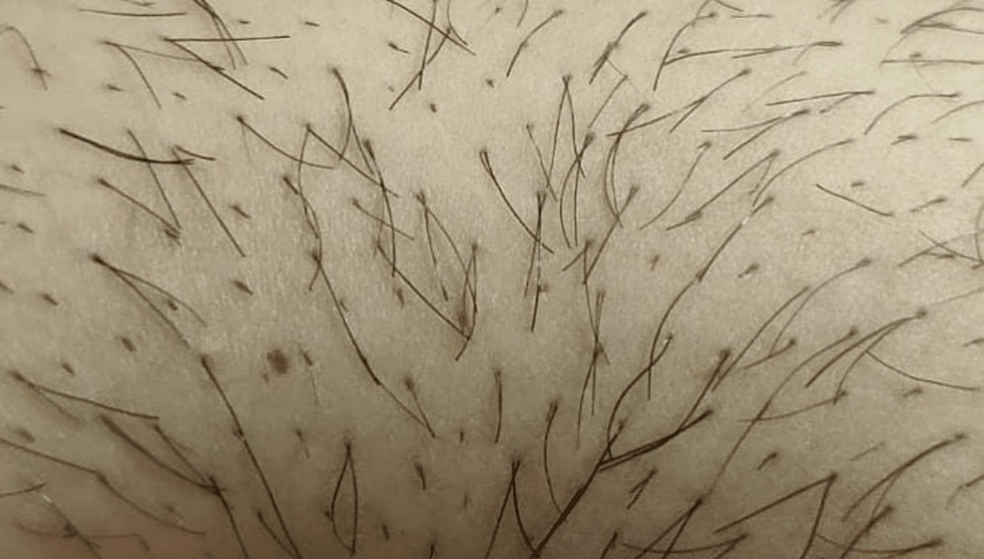
Generalized pili mulitgemini

A 25-year-old male presented with complaints of mild pain and swelling localized to an area on the lower part of the abdomen for one day. He has had multiple similar episodes for more than six years which resolved without intervention. Physical examination demonstrated the presence of a well-circumscribed erythematous and fluctuant pustule measuring 1 cm x 1 cm with two shafts of hair arising from a single hair follicle from its center as can be seen in Figure 1. The follicular ostia was free of scarring. The results were consistent with folliculitis. He had mentioned the presence of generally thick body hair with thick hair shafts. The patient was diagnosed with pili multigemini which was widespread on the entire abdomen (Figure 2) and was discharged on topical antibiotics and offered electrolysis but he refused. On follow-up, the patient reported resolution of the folliculitis with topical antibiotics.

## Discussion

The etiology and origin of pili multigemini is not very well understood. In 1883, Flemming had suggested that the condition occurs due to the splitting of a single papilla or merging of different papillae. But in 1892, Giovanni proposed that it was caused by the reactivation of the silent embryonic epithelial germ cells [2]. Pinkus proposed that the abnormal splitting of a single dermal papilla into multiple papillae may be the etiology of the condition [1]. A recent study has highlighted the importance of the bulge area behind the etiology of the condition. The bulge area acts as a source for new follicle generations and can be predisposed to chemical carcinogenesis during the anagen stage. They also propose that mechanical trauma and inflammatory changes can be the stimulus for the multiplication of stem cells inside the bulge [5].

It has been found to be associated with alopecia areata of the beard and inflammatory nodules, however, the etiology behind it has not yet been discovered [1,6]. There has also been one report of the association of pili multigemini with cleidocranial dysostosis [6]. It is important to differentiate it from compound hairs. Compound hairs have separate hair follicles which merge in the superficial layers of the dermis, giving the appearance of multiple hair shafts originating from a single follicle [1]. 

Combination immunotherapy with dabrafenib and trametinib is known to cause cutaneous adverse effects. Dysplasias involving the hair shaft have not been reported, however, Avila et al. detected pili multigemini in a patient treated with this immunotherapy and hypothesized a genetic link or activation of the silent germ cells behind the etiology of the condition [7]. Pili multigemini has also been documented after hair transplantation and it has been suggested to occur most likely due to trauma to the hair follicle and papilla [8]. 

Pili multigemini can be detected by trichoscopy, a non-invasive and fast technique that can be used to see the hair’s morphology which may go unnoticed by the naked eye. Trichoscopy can provide information about the activity and severity and can also be used to monitor the progression after treatment. It helps avoid invasive or painful procedures such as skin biopsy or plucking of hair [4]. 

Cambiaghi et al. suggest the use of scanning electron microscopy (SEM) as an alternate method to diagnose pili multigemini. SEM can show the presence of each dividing hair with its respective cuticle which can help to distinguish pili multigemini from trichoptilosis. It can also be used to distinguish pili multigemini from pili bifurcati, wherein the hair shaft splits to form parallel branches before eventually rejoining [2]. Electrolysis and laser ablation can be offered in symptomatic patients as a form of treatment [4, 9]. 

One research has demonstrated folliculitis in the beard that was managed with laser hair removal and showed improvement in symptoms of pili multigemini [10]. On the other hand, another research showed the development of folliculitis after treatment of pili multigemini with Nd:YAG (neodymium-doped yttrium aluminum garnet) laser treatment [9].

## Conclusions

Pili multigemini is often asymptomatic and hence may be under-reported. A thorough inspection and the use of trichoscopy can aid in the diagnosis and subsequent therapy can be offered. The available data may not represent the actual incidence rate. Further studies can be conducted to determine its prevalence. The speculated genetic link behind its occurrence can also be explored.
